# Practical continuous-variable quantum key distribution with squeezed light

**DOI:** 10.1126/sciadv.aed4162

**Published:** 2026-10-16

**Authors:** Huy Q. Nguyen, Ivan Derkach, Hou-Man Chin, Adnan A.E. Hajomer, Akash nag Oruganti, Radim Filip, Ulrik L. Andersen, Vladyslav C. Usenko, Tobias Gehring

**Affiliations:** ^1^ Center for Macroscopic Quantum States (bigQ), Department of Physics, Technical University of Denmark, 2800 Kongens Lyngby, Denmark.; ^2^ Department of Optics, Faculty of Science, Palacky University, 771 46 Olomouc, Czech Republic.; ^3^ Celare Quantum Communications ApS, 2000 Frederiksberg, Denmark.

## Abstract

Continuous-variable quantum key distribution (CV-QKD) has gathered significant interest for its potential to achieve high secret key rates and seamless integration with existing optical communication infrastructure. State-of-the-art CV-QKD systems primarily use coherent states for simplicity. However, squeezed states of light have been theoretically shown to offer significant advantages, including higher secret key rates, greater resilience to excess noise, and reduced requirements on information reconciliation efficiency. In this work, we experimentally verify these theoretical predictions and propose and demonstrate a practical squeezed-state CV-QKD system based on modern local-local oscillator and digital signal processing techniques. Operating over fiber channels and considering finite-size security against collective attacks, we show the advantages of our system over its coherent-state counterpart. Our work paves the way for squeezed states to become practical resources for quantum key distribution and other quantum information protocols.

## INTRODUCTION

Quantum key distribution (QKD) enables information-theoretic secure communication based on the principles of quantum physics (*1*). Among the different QKD flavors, continuous-variable QKD (CV-QKD) encodes information onto the quadratures of the optical field (*2*). Recent advances have established CV-QKD with practical composable security (*3*), enabling extended operational distances (*4*, *5*) and increased secret key rates (*6*).

Squeezed states form a foundational resource for numerous quantum information protocols. In quantum computation, they can be used for blind quantum computing (*7*) and universal quantum computation with cluster states (*8*), while recent experimental advances also demonstrate a quantum learning advantage (*9*). In quantum metrology, squeezed states have been shown to enable extreme sensitivity beyond the standard quantum limit and the ideal NOON state limit (*10*) and to enable distributed quantum sensing with entangled probes (*11*).

In the realm of quantum communication, squeezed states can be leveraged to implement oblivious transfer (*12*) and in CV-QKD to extend transmission distances (*13*), enable advanced security (*14*), and eliminate information leakage in purely lossy channels (*15*). In quantum state discrimination, squeezed states can be used to surpass the coherent Helstrom bound (*16*, *17*), enabling advancements in quantum cryptography and quantum communication. Given their versatility and central role in these applications, squeezed states are a strong candidate for resource states in the emerging quantum internet, enabling additional functionalities beyond QKD, especially since they are crucial for continuous variable (CV) entanglement swapping (*18*), quantum teleportation (*19*, *20*).

State-of-the-art CV-QKD systems typically use coherent signal states generated by laser sources, offering simplicity in implementation (*21*, *22*). An alternative approach is to encode information onto quadrature-displaced squeezed states (*23*), which was the initial proposal of Gaussian CV-QKD. The most inherent advantage of squeezed states is the increase in signal-to-noise ratio (SNR) due to the lower noise variance, which leads to higher mutual information (MI). Furthermore, it has been shown that squeezed states minimize the information leakage and possibly completely remove the eavesdropper’s information (*15*, *24*). This make them extremely robust for free-space and satellite QKD (*25*, *26*). Theoretical studies have suggested that using squeezed states can allow CV-QKD systems to tolerate higher noise levels, to extend transmission distances, and to enhance the performance of systems with low information reconciliation efficiency (*25*, *27*, *28*). Despite these benefits, generating and detecting squeezed states presents additional complexity with previous experimental demonstration of squeezed states in CV-QKD (*13*–*15*) were performed in laboratory conditions over short free-space channels and emulated the channel loss. Thus, they are quite far from full-scale implementation, enabling deployment in optical fiber infrastructure. An important step toward this goal is to demonstrate the advantage of QKD with squeezed states in a practical setting.

Substantial progress has been made on developing compact squeezed sources (*29*) and implementing them on photonic integrated chips (*30*–*33*). These developments paved the way for cheap and portable squeezed light sources. However, transmitting quadrature-squeezed states through an optical fiber and remotely detecting them with a homodyne detector remains challenging. Two major challenges to fiber-based squeezed-state CV-QKD are the phase shifts introduced by the fiber channel and the necessity for locally generated local oscillators (LLOs) to avoid security loopholes (*34*, *35*). Efforts to address these issues using optical phase-locked loops (OPLLs) (*36*) have encountered limitations, such as phase drift that destabilizes quadrature variances and correlations, complicating reliable key extraction (*37*). Furthermore, an OPLL undoubtedly increases the system’s overall complexity. Because of the above-described practical challenges, squeezed states were not, until now, considered to be a realistic competitor of coherent states in CV-QKD.

Over the past decade, digital signal processing (DSP) has served as a powerful workhorse for modern coherent-state CV-QKD systems (*38*–*41*). Leveraging techniques that have matured over more than half a century with digital computing and four decades alongside the internet, DSP has substantially improved both the performance and ease of implementation of these systems. Recently, DSP has also been applied to enable the reconstruction of squeezed states and facilitate passive CV-QKD over deployed fiber (*42*). Aside from the requirement of a squeezed-light source, this advancement allows squeezed-state CV-QKD to operate with simplified optical subsystems comparable to those of conventional coherent-state protocols.

In this work, we propose, experimentally demonstrate and theoretically analyze a prepare-and-measure CV-QKD protocol using quadrature-modulated squeezed states over optical fiber and effectively measured by radio frequency (rf) heterodyne detection. Through DSP, we eliminate the need for optical frequency and phase locking, thus enhancing the practicality of the protocol. We perform a security analysis taking into account finite-size effects and compare it with the conventional coherent states protocol under the same condition. Our results reveal a substantial enhancement in secret key rate with squeezed states compared to coherent states. Our analysis further shows that the squeezed-state CV-QKD protocol performs exceptionally well with lower reconciliation efficiency, reducing the computational demands of reverse reconciliation—a critical bottleneck in CV-QKD—thus promising a low-complexity, real-time and high-speed key generation system. Last, we compare squeezed-state and coherent-state protocols under increasing excess noise levels, demonstrating that squeezed states are notablely more resilient. This resilience makes squeezed-state CV-QKD an attractive candidate for multiplexing with high-power classical channels in existing optical telecom infrastructure.

## RESULTS

### Practical squeezed-state CV-QKD protocol

The squeezed-state CV-QKD protocol is illustrated in Fig. 1. Unlike coherent states, squeezed states are never perfectly pure in practice, posing a security risk as uncharacterized impurities may be exploited by an eavesdropper (*25*, *28*). While impurity can be incorporated into the security analysis, it reduces performance and still relies on accurate squeezing level monitoring, risking underestimation of eavesdropper influence. Alternatively, the continuous monitoring of both squeezing and purity allows treating the additional noise as trusted preparation noise. Since the most conservative security analysis assumes maximum added noise (*28*), all preparation noise can be attributed to the anti-squeezed quadrature, represented as Δ*V*_AN_.

**Fig. 1.**
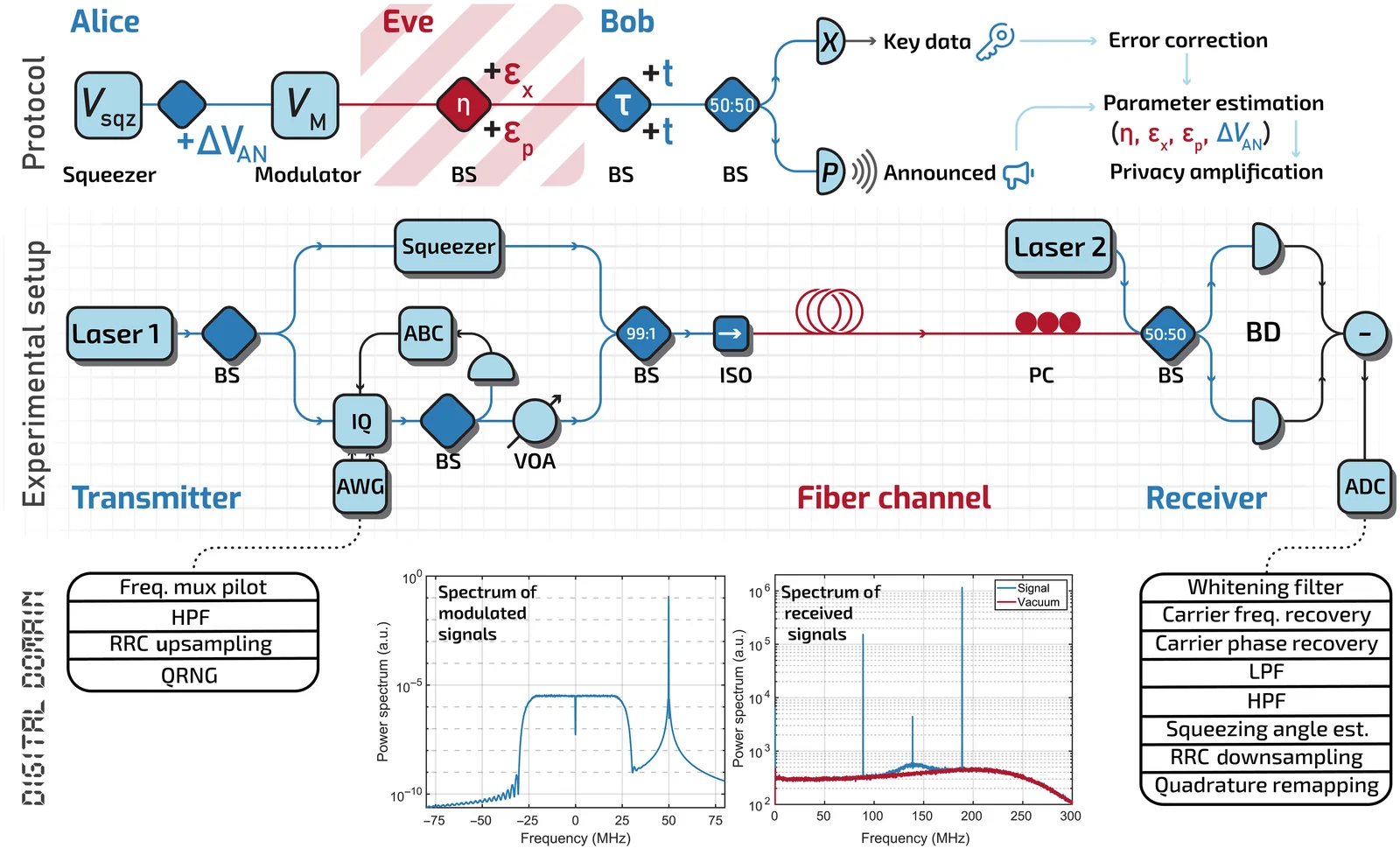
Protocol. Alice generates squeezed vacuum states with variance *V*_sqz_ in *x* quadrature and $1/V_{\text{sqz}}+\Delta V_{\text{AN}}$ in *p* quadrature and displaces them in both quadratures (modulation variance *V*_M_). Eve’s channel subjects the signal to losses η and quadrature dependent noise with variance $\epsilon_{x\left(p\right)}$. Bob performs heterodyne detection on the signal with an efficiency τ and added electronic noise *t*. The measurement outcomes of the *p* quadrature are publicly announced and fully used for parameter estimation. The measurement outcomes of the *x* quadratures are used to generate the keys. Here, we assume that we do error correction before parameter estimation (*3*, *66*). Experimental setup: IQ, in-phase and quadrature modulator; AWG, arbitrary waveform generator; ABC, automatic bias controller; VOA, variable optical attenuator; ISO, optical isolator; PC, polarization controller; BD, balanced detector; ADC, analog-to-digital converter; RRC, root-raised-cosine; QRNG, quantum random number generator; HPF, high-pass filter; LPF, low-pass filter.

Early theoretical works (*23*, *27*, *43*, *44*) on squeezed-state CV-QKD did not address signal-state verification in practical settings. Traditional protocols switch quadratures for squeezing and key encoding, with homodyne measurements contributing to either the raw key or state monitoring. Although this approach theoretically performs well and tolerates high noise (*13*, *25*, *27*), it requires careful resource allocation in the finite-size regime (*28*). Measuring both quadratures simultaneously allows continuous key generation and real-time state verification (*44*), enhancing security at the cost of reduced detection efficiency and added vacuum noise—a cost that can be partially mitigated by publicly disclosing measurements of the antisqueezed quadrature. The security analysis of such protocol is based on the three-mode entanglement-based purification model detailed in (*27*, *28*) and the Supplementary Materials. In the asymptotic regime, the secret key fraction (SKF) secure against collective attacks is given by

$$K_{\infty }=\text{max}\left\{0,\beta \;I\;\left(A,B\right)-\chi \;\left(B:E\right)\right\}$$ (1)

where β is the reconciliation efficiency, I(A,B) is the MI between Alice and Bob in the squeezed quadrature, and χ(B:E) is Holevo bound—the upper bound on the accessible information of Eve on homodyne measurement results used for the key generation. The measurements and public disclosure of the antisqueezed quadrature aids parameter estimation (see the Supplementary Materials) and partially reduces the entropy of eavesdroppers state bounded by χ(B:E), which now becomes conditioned on the announcement of the nonsignal quadrature *B^P^*. The Holevo information χ(B:E) reads

$$\chi \left(B:E\right)=S\left(\rho_{{\mathrm{AB}}^{X}|B^{P}}\right)-S\left(\rho_{A|B^{X}B^{P}}\right)$$ (2)

This approach has been shown to be more efficient than conventional homodyne-based protocol, especially in high-noise quantum channels, and for smaller block sizes (*28*). For further details on the evaluation of respective elements of the key rate, see Eq. 1, and for the purification model of the protocol, see (*28*).

Usually, detector efficiency is not considered in parameter estimation. However, detector inefficiency reduces correlations between Alice’s and Bob’s data, which are essential for accurately estimating the channel transmission. This inefficiency consequently deteriorates the quality of the transmission estimate. Therefore, we extend the parameter estimation from (*28*) to explicitly include detector efficiency.

The variance of the transmission estimator, taking detector efficiency into account, is given by

$$\text{Var}\left[\hat{\eta }\right]=\sigma_{\eta }^{2}\approx \frac{\eta \left[V_{\text{Np}}^{\prime }+4\eta V_{M}+V_{\text{Nx}}^{\prime }\right]}{2{\mathrm{nV}}_{M}}$$ (3)

Here

$$V_{\mathrm{Nx}}^{\prime }=\frac{2}{\tau }+\eta \left(\epsilon +V_{\text{sqz}}-1\right)+\frac{1-\tau }{\tau }\left(V_{D}-1\right)$$

$$V_{\text{Np}}^{\prime }=\frac{2}{\tau }+\eta \left(\epsilon +\frac{1}{V_{\text{sqz}}}+\Delta V_{\text{AN}}-1\right)+\frac{1-\tau }{\tau }\left(V_{D}-1\right)$$

The variance of the excess noise estimator in the X and P quaratures reads

$$\text{Var}\left[{\hat{\epsilon }}_{x}\right]=\sigma_{{\hat{\epsilon }}_{x}}^{2}\approx \frac{2}{n}{\left[V_{\text{Nx}}^{\prime }\right]}^{2}+{\left(1-V_{S}\right)}^{2}\text{Var}\left[\hat{\eta }\right]$$ (4)

$$\text{Var}\left[{\hat{\epsilon }}_{p}\right]=\sigma_{{\hat{\epsilon }}_{p}}^{2}\approx \frac{2}{n}{\left[V_{\text{Np}}^{\prime }\right]}^{2}+{\left(1-1/V_{S}-\Delta V_{\text{AN}}\right)}^{2}\text{Var}\left[\hat{\eta }\right]$$ (5)

The SKF taking into account finite-size effect reads

$$K_{\text{finite}}=K_{\infty }\left(\eta_{\text{low}},\epsilon_{\text{up}}\right)-\Delta (n)=$$ (6)

$$\text{max}\left\{0,\beta I\left(\eta_{\text{low}},\epsilon_{\text{up}}\right)-\chi \left(\eta_{\text{low}},\epsilon_{\text{up}}\right)-\Delta \left(n\right)\right\}$$ (7)

here, Δ(*n*) is the asymptotic equipartition property correction term, which quantifies the finite-size penalty associated with replacing the smooth conditional min-entropy by its asymptotic conditional von Neumann entropy. This term vanishes in the limit n→∞. The quantity *n* denotes the number of signals used for key extraction. Channel efficiency $\eta_{\text{low}}=\eta -6.5\sigma_{\hat{\eta }}$ and the excess noise $\epsilon_{\text{up}}=\epsilon +6.5\sigma_{\hat{\epsilon }}$ are the worst case estimation of the corresponding to the estimation error probability of 10^−10^. Note that the excess noise here is the higher value of the two quadratures. Detail analysis of squeezed state CV-QKD taking into account finite-size effect under collective attacks can be found in (*28*).

### Squeezed states under imperfect reconciliation

In CV-QKD, information reconciliation remains a critical bottleneck for real-time key generation. Since the SNRs are typically far below those of classical communication, error correction becomes a challenging task (*45*). In practice, pushing the error correction code rate to approach the channel capacity yields high reconciliation efficiency but severely increases the frame error rate (FER) at low SNRs. As the reconciliation efficiency approaches unity, the code operates closer to the Shannon limit. This reduces the gap between the code rate and the channel capacity, leaving less tolerance for finite-size effects, decoder imperfections, and statistical fluctuations. As a result, the FER may increase sharply in practical implementations. Moreover, maintaining this high efficiency requires hundreds of decoder iterations, which imposes a massive computational burden. Squeezed states provide an inherent advantage in this regard, as their lower noise variance leads to higher SNR and, in turn, higher MI (see fig. S2). A more detailed discussion of the information-theoretic reasons behind the robustness of squeezed states can be found in the Supplementary Materials.

To experimentally demonstrate the advantage of using squeezed states over coherent states in CV-QKD, we implemented both protocols over a 50-km ultra-low-loss (ULL) optical fiber with a total loss of 7.25 dB. Experimental data were gathered from 250 frames, each composing of $\approx 4\times 10^{5}$ quantum symbols, resulting in $\approx 10^{8}$ quantum symbols. The parameters estimated from the experiments are shown in Table 1. The estimated squeezing variance was 0.417 SNU with the antisqueezed noise $\Delta V_{\text{AN}}=3.029$ SNU. The modulation variance was set to 1.372 SNU. Here, the excess noise ϵ is shown at the input of the channel, leading to $\epsilon_{i}=2u_{i}/\left(\eta \tau \right),\;\text{for}\;i=x,p$, where *u_i_* is the excess noise at detection and τ = 0.68 is the trusted detection efficiency (see the Supplementary Materials).

**Table 1.** Parameters estimated from measurements with 50-km fiber channel. The parameters were estimated from 10^8^ quantum symbols.

	*V*_sqz_ (SNU)	Δ*V*_AN_ (SNU)	*V*_M_ (SNU)	$\epsilon_{x}$ (SNU)	$\epsilon_{p}$ (SNU)	η	*V*_D_ (SNU)
Sqz.	0.417	3.029	1.372	0.041	0.037	0.166	1.07
Coh.				0.032	0.031	0.163	1.07

Error correction was performed with a multidimensional information reconciliation scheme implementing a multiedge-type low-density parity check (MET-LDPC) error correction code (*45*). The base code rate of the original MET-LDPC code was 0.02, and puncturing was used to adjust the code rate to accommodate varying reconciliation efficiencies (*45*). Given a codeword length *n*, information bits *k*, and puncturing length *p*, the post-puncturing code rate is given by $R_{\text{punc}}=k/\left(n-p\right)$ (*45*), which yields a code efficiency of $\beta =R_{\text{punc}}/I\left(A:B\right)$, where I(A:B) is the MI of the classical information held by Alice and Bob after the quantum phase of the protocol. The parameters used for error correction are shown in tables S1 and S2 for the coherent states protocol and the squeezed-state protocol, respectively.

When accounting for the FER of the error correction process, the finite-size SKF (Eq. 6), expressed in bits per quantum symbol (bits/symbol), is given by

$$K_{\text{finite}}=\left(1-\text{FER}\right)\left[R_{\text{punc}}-\chi \left(\eta_{\text{low}},\epsilon_{\text{up}}\right)-\Delta \left(n\right)\right]$$ (8)

In this analysis, the MI was calculated on the basis of the discretized quantum symbols held by Alice and Bob.

Figure 2A shows the results where the estimated SKF is plotted versus the error correction efficiency. The green bubbles represent the FER of the error correction process. The yellow bubbles indicate the minimal number of decoder iterations (ITR) for which we achieved the FER written in the green bubbles. Here, we did not aggressively minimize the number of decoder iterations as this would not make sense in a practical setting, where the FER is also dependent on statistical fluctuations. Furthermore, the decoder implements early termination based on a syndrome check. As expected the number of required decoder iterations decreases with decreasing code efficiency as the code is further away from the Shannon capacity of the channel. The solid and dashed lines display the calculated SKF assuming an FER of zero. For high efficiencies, our experimental data points deviate from these lines as the FER becomes substantially larger than 0. Note that the decoder is optimized for the mean MI, so deviations from this operating point, caused, for example, by phase noise or residual antisqueezed noise, can increase the FER sharply, especially when operating close to the Shannon limit.

**Fig. 2.**
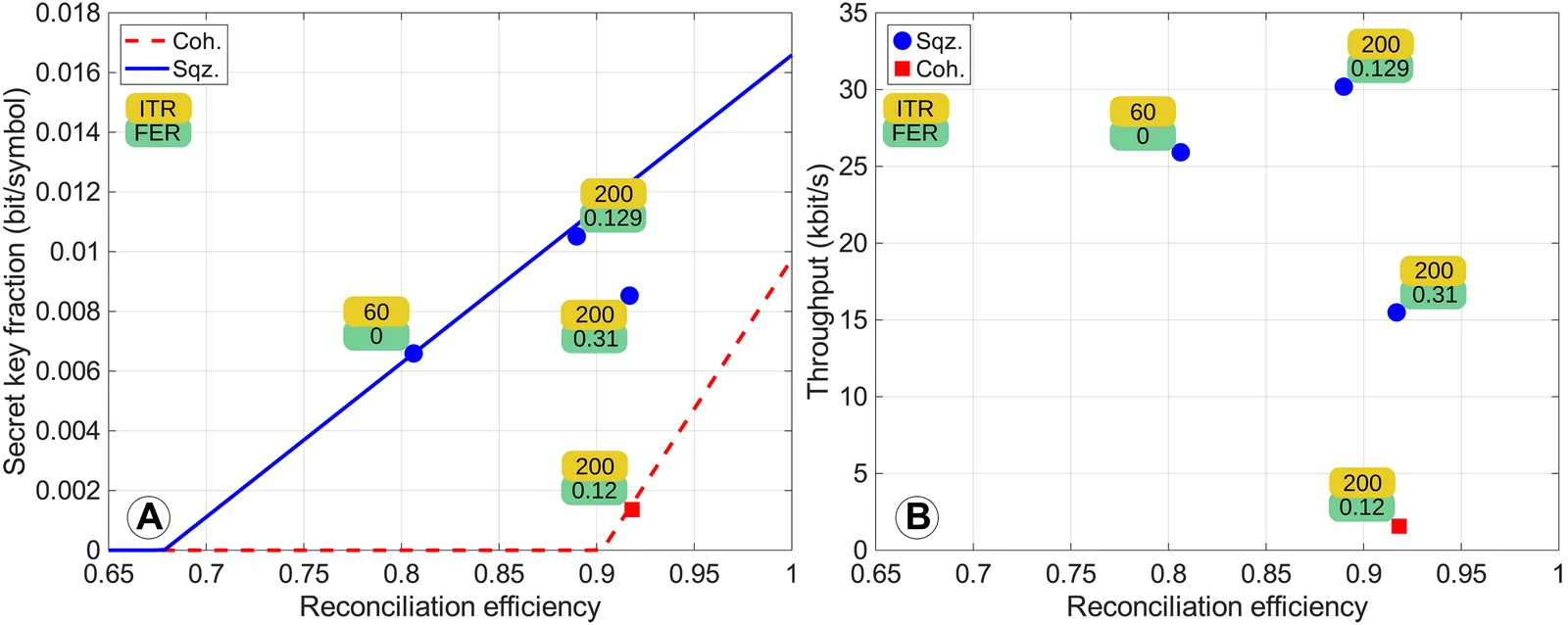
Key generation after 50-km fiber when varying reconciliation efficiency. (**A**) SKF in bits per quantum symbol (**B**) Secret key throughput excluding DSP time.

As seen in the figure, the performance degradation in the coherent-state protocol is more pronounced as the β decreases, while the squeezed-state protocol is less affected by this reduction. This experimentally demonstrates the theoretical prediction (*27*) that the SKF scales differently with reconciliation efficiency for the two protocols. At a high reconciliation efficiency of β≈0.92, the squeezed-state protocol achieves an SKF of 0.0085 bits/symbol, more than six times higher than the coherent states protocol, which yields 0.0014 bits/symbol. As β is relaxed, both the FER and the required number of iterations decrease. This flexibility enables the squeezed-state protocol to achieve a peak SKF of 0.0105 bits/symbol at β≈0.89%, whereas the coherent states protocol cannot generate finite-size keys when β < 0.9.

The performance of CV-QKD systems is often limited by information reconciliation throughput. This throughput is inversely proportional to the number of decoder iterations. Given the lower efficiency of codes with fewer decoder iterations (Fig. 2A), a new trade-off must be considered. We tested this by observing the throughput of an information reconciliation implementation on an NVIDIA GPU (2048 cores, 1.3 GHz), with results shown in Fig. 2B. Compared to Fig. 2A, we observed the optimal reconciliation efficiency (β) for finite-size throughput, which peaked at β≈0.89 for squeezed states. At this optimum, the advantage of squeezed states was evident, yielding a throughput of 30.19 kbit/s, nearly 20 times greater than the 1.57 kbit/s produced by the coherent state protocol. Note that given the same number of decoder iterations, the coherent state protocol’s decoding time is about double that of the squeezed state protocol, as the latter generates a key from only the squeezed quadrature, while the former uses both.

### Squeezed states under high excess noise

Excess noise is another major bottleneck that directly degrades CV-QKD performance (*4*). An increase in noise leads to an increase in information leakage to the eavesdropper. Squeezed states have been shown to be very effective at minimizing Holevo information (*15*, *44*). Since squeezed-state protocol only uses one quadrature to generate keys (compared to two for the coherent states), the question becomes how fast Holevo information grows with increasing excess noise. The key intuition behind the advantage of squeezed states under high excess noise is that their information leakage grows much more slowly than that of coherent states. A more detailed analysis of the information-theoretic basis behind the squeezed-state protocol’s robustness against excess noise can be found in the Supplementary Materials (see fig. S2C).

To demonstrate the noise tolerance advantage of squeezed states in CV-QKD, we introduced controlled noise during modulation and tested the system across two different channel lengths. Experimental data were gathered from 250 frames, each composing of $\approx 4\times 10^{5}$ quantum symbols, resulting in $\approx 10^{8}$ quantum symbols. Theoretical simulations (solid and dashed lines in Fig. 3) were conducted under conditions of fixed channel attenuation for each scenario. Here, we assumed 92% reconciliation efficiency with FER=0. Tables S3 and S4 in present details on the parameter estimations and SKF for the squeezed and coherent state protocols at various excess noise levels. For the squeezed-state protocol, the noise floor without induced excess noise is estimated rather than directly measured, as explained in the Supplementary Materials, and the noise across both quadratures is conservatively symmetrized to the higher value. As a result the squeezed-state protocol generally shows higher intrinsic noise levels than the coherent states protocol.

**Fig. 3.**
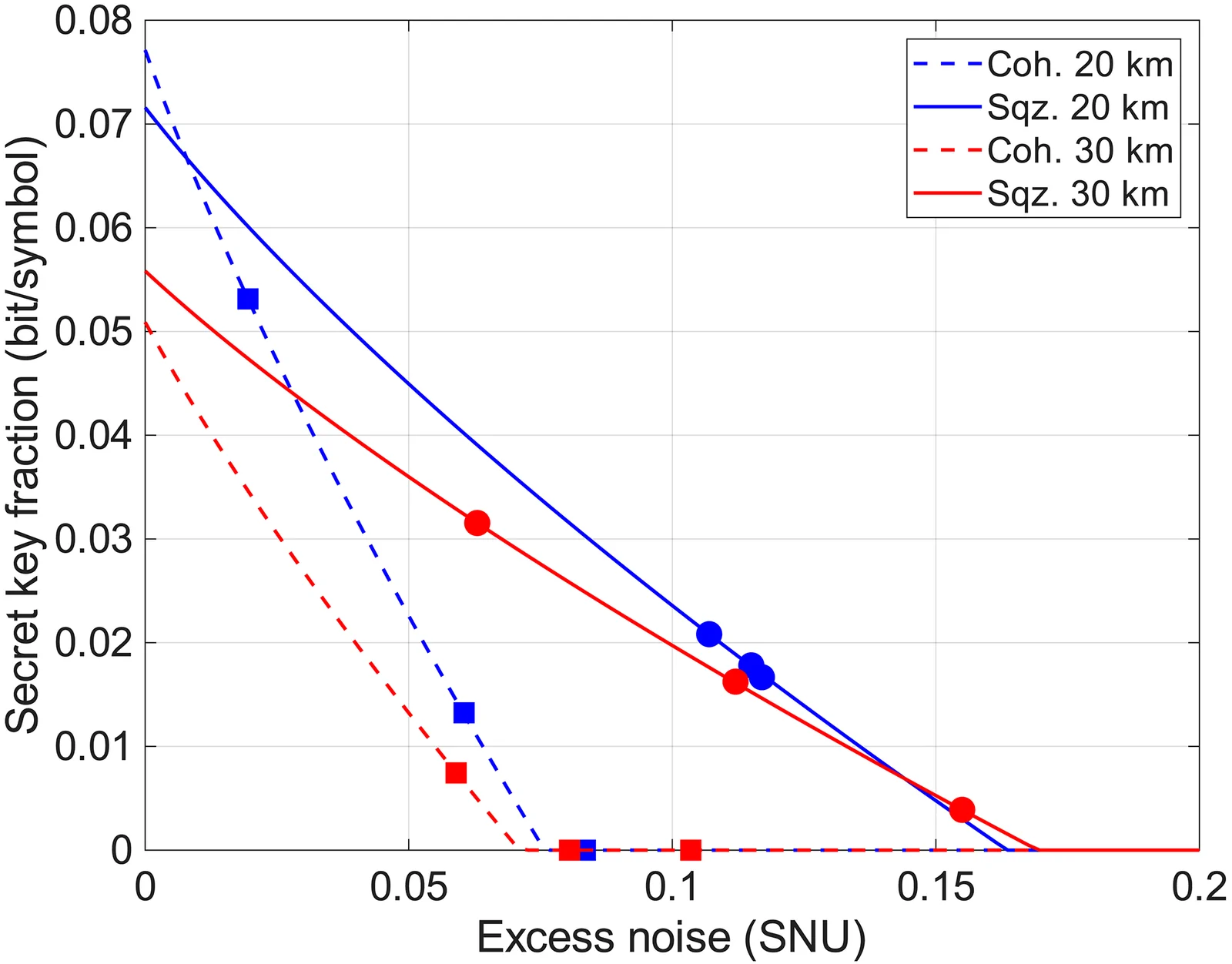
SKF after 20- and 30-km fiber at different levels of excess noise.

Figure 3 shows the experimental results and a fitted theoretical model. It is visible that the two protocols have a different scaling in key rate when excess noise is increased. Starting at low excess noise levels, we can see from the theory curves that the coherent states outperform squeezed states because squeezed states only use the squeezing quadrature to generate the keys. However, as noise increases, squeezed states begin to demonstrate superior performance. For instance, for the 20-km channel, at ≈117 mSNU of excess noise, the squeezed-state protocol achieves an SKF of 0.0167 bits per symbol, whereas the coherent states protocol, at a lower noise level of ≈61 mSNU, it only achieves 0.00132 bits per symbol and fails to produce a finite-size positive secret key above 84 mSNU of excess noise.

This trend is even more pronounced over the 30-km channel, where squeezed states consistently outperform coherent states across all experimental excess noise levels. At a lower excess noise level (just above 60 mSNU), the squeezed-state protocol achieves a finite-size key rate of 0.0315 bits per symbol, more than four times the rate of the coherent states protocol at 0.0074 bits per symbol. As excess noise increases, the coherent states protocol cannot tolerate around 80 mSNU, whereas the squeezed-state protocol can generate secret keys at noise levels exceeding 175 mSNU.

From the theory curves, we find that the QKD protocol operating over the 30-km channel tolerates more noise than over the 20-km channel. This is due to differences in both the anti-squeezed noise and the modulation variance between the two distances, with the 30-km channel’s modulation variance being closer to its optimal value.

## DISCUSSION

The benefits of using squeezed states in CV-QKD have been explored extensively in prior theoretical studies (*27*, *28*). While notable experiments have been conducted with channel loss simulated via optical attenuators, implementing CV-QKD systems using squeezed states over actual fiber channels remains challenging due to the complexity of generating and detecting squeezed states. In this work, we present the first practical demonstration of a CV-QKD system using squeezed states transmitted over fiber channels. By incorporating a specialized DSP chain, we substantially simplify the application of squeezed states in CV-QKD, preserving several key advantages over coherent-state protocols.

First, our experimental results show that the squeezed-state protocol outperforms the coherent-state protocol, particularly under conditions of low reconciliation efficiency. To achieve high reconciliation efficiency the decoder requires many iterations, reducing its throughput. Squeezed states help alleviate the strain on classical postprocessing, paving the way for high-speed, real-time key generation in CV-QKD systems.

Second, we demonstrated that squeezed light can improve CV-QKD systems’ tolerance to excess noise. With QKD systems reaching closer to end users, it becomes necessary to multiplex the quantum signal with strong classical light. CV-QKD has long been considered to be a good contender for this. With the ability to maintain high performance in noisy channels, squeezed light promises to be a valuable resource when integrating CV-QKD systems into existing optical telecom networks.

Recent efforts to increase the speed and practicality of CV-QKD systems entail the use of discretely modulated (DM) protocols. DM CV-QKD can reduce the stringent bit-resolution requirements of digital systems, thereby reducing implementation costs and increasing operating speeds while also reducing the complexity of classical postprocessing (*6*, *46*, *47*). Expanding the application of discrete modulation from coherent states to squeezed states offers a promising avenue to enhance systems performance and further exploit the inherent advantage of the nonclassical states. However, theoretical techniques need to be improved to expand to DM squeezed states especially in practical setting.

Beyond conventional fiber channels, free-space CV-QKD serves as a natural alternative when fiber-optic infrastructure is either unavailable or impossible to deploy securely (*48*, *49*). Furthermore, satellite-based quantum communication offers an appealing route toward a global-scale, intercontinental quantum network (*50*, *51*). Theoretically, squeezed states are proven to substantially enhance the robustness of CV-QKD against high channel loss, modulation noise, and atmospheric turbulence (*25*, *26*). Although the distribution of squeezed light through atmospheric channels has been previously demonstrated, it relied on complex locking schemes (*52*). The simplicity and practicality of our proposed method make it directly applicable to free-space channels, thus opening a promising avenue for enhancing free-space CV-QKD performance.

The technique developed in this work could also benefit other quantum information and quantum metrology applications. Beyond QKD, quantum secret sharing (QSS) enables the secure distribution of information among multiple parties, provided a sufficient number of them remain honest (*53*, *54*). While QSS has been demonstrated using discrete variables (*55*, *56*), recent advancements include source device–independent protocols that are robust against coherent attacks (*57*). In the CV domain, QSS has been realized with entangled states (*58*) and, more recently, through practical implementations using coherent states (*59*) and LLOs (*60*). The modulated squeezed states with techniques outlined in this paper can serve as a key resource to enhance the performance of these practical CV-QSS protocols.

Furthermore, squeezed states have long been shown to provide notable enhancements in metrology. For instance, the scheme in (*11*) demonstrated that displaced single-mode squeezed states can achieve distributed phase sensing beyond the standard quantum limit. However, that work was a proof-of-concept experiment involving free-space distribution on an optical table with a simulated phase shift. Moreover, many QSS protocols have been shown to benefit greatly from techniques in quantum metrology (*61*). Our method can be used to extend these ideas to deployed fiber networks, thus substantially expanding the practicality and reach of the technologies.

Because of implementation challenges, squeezed-state CV-QKD has not been a major focus of active research, with most previous work limited to theoretical analyses or proof-of-concept experiments. Our work demonstrates that modern techniques from coherent-state CV-QKD systems can be effectively extended to squeezed states. Combined with recent advances in photonic integration, our results position squeezed states as a strong candidate not only for CV-QKD but also for broader quantum information applications. These findings highlight the need for renewed focus and exploration in this promising area.

## MATERIALS AND METHODS

Figure 1 shows the experimental setup as well as the DSP procedure. On the transmitter side, squeezed light was generated at 1550 nm via spontaneous parametric down-conversion using a periodically poled potassium titanyl phosphate crystal in a hemilithic cavity. Details about the squeezed light source are available in (*29*). Alice draws the displacement α of the quantum symbols with Gaussian statistics using a quantum random number generator. The system operated at a rate of 50 MBaud. The quantum symbols were then upsampled using a root-raised cosine pulse shape with a roll-off factor of 0.2. Baseband modulation was chosen to fully use the squeezing bandwidth. A fourth-order Butterworth high-pass filter with a cutoff frequency of 170 kHz was applied to ensure the same temporal shaping as the mode detected by the receiver (*62*, *63*). A 50-MHz pilot tone was frequency-multiplexed to serve as a phase reference for the carrier recovery. The modulation waveform was used to drive an IQ modulator, and the optical signal strength was controlled with a variable optical attenuator. The modulated signal was combined with the squeezed light at a 99:1 beam splitter, displacing the squeezed vacuum state in phase space with Gaussian statistics. An optical isolator was installed in the path to prevent back reflections from reaching the squeezed light source. The squeezed light source can be viewed as a modular add-on to a conventional coherent-state system, enabling the implementation of squeezed-state CV-QKD without major modifications. When benchmarking our proposed protocol against the coherent-state protocol, the switch simply requires physically blocking the squeezed light from the free-space cavity.

At the receiver side, a separate free-running laser was used to generate a LLO. The quantum signal was measured using rf heterodyne detection with a balanced detector by frequency detuning the receiver laser with respect to the transmitter laser by an amount larger than the squeezing bandwidth (*42*). After the measurement, we digitally reconstructed the displaced squeezed states as we describe in the following.

First, the frequency difference between the two lasers was estimated and corrected using the analytic representation of the pilot tone. After frequency offset compensation, we performed carrier phase recovery using an unscented Kalman filter (*39*). To reduce out-of-band noise, particularly the high-power pilot tone, we applied a low-pass filter around the quantum signal. The baseband modulation contained unwanted low-frequency components, such as the unsuppressed optical carrier and the automatic bias controller dither signal, which we eliminated using a high-pass filter (*62*). Following carrier phase recovery, we corrected the phase noise between the two lasers. However, the recovered quadratures did not necessarily align with the squeezing and antisqueezing quadratures. Because of the intrinsic asymmetry of the squeezed state ensemble, any phase misalignment produces correlations between the X and P quadratures. By minimizing these correlations, we identified the optimal rotation needed to align Bob’s quadratures with the squeezing and antisqueezing quadratures (*42*). However, because the squeezing field was not locked to the modulated coherent states, there is still a mismatch between Alice’s modulation quadratures and Bob’s measured quadratures. In coherent-state systems, quadrature remapping has been used to rotate the received symbols, maximizing the covariance between Alice’s and Bob’s symbols (*64*). We applied this technique to Alice’s symbols, recovering both the modulation angle and the covariance between Alice’s and Bob’s states. This method is applicable only under the conditions of symmetric modulation—where the modulation variance is identical across both quadratures—and equal detection efficiencies for both quadratures of the optical field.

## Supplementary Materials

### This PDF file includes:

Supplementary Text Figs. S1 to S3 Tables S1 to S4 References
