# Supplementary material for: Practical continuous-variable quantum key distribution with squeezed light
Source: Sci Adv. 2026 Oct 16;12(42):eaed4162. doi: 10.1126/sciadv.aed4162 (PMC13674495; doi:10.1126/sciadv.aed4162)
Supplement: Supplementary file 1 [file sciadv.aed4162_sm.pdf]

Science Advances
AAAS

Supplementary Materials for

Practical continuous-variable quantum key distribution with squeezed light

Huy Q. Nguyen *et al.*

Corresponding author: Huy Q. Nguyen, hqung@dtu.dk; Tobias Gehring, tobias.gehring@fysik.dtu.dk

Sci. Adv. **12**, eaed4162 (2026)
DOI: 10.1126/sciadv.aed4162

This PDF file includes:

Supplementary Text
Figs. S1 to S3
Tables S1 to S4
References

Security model

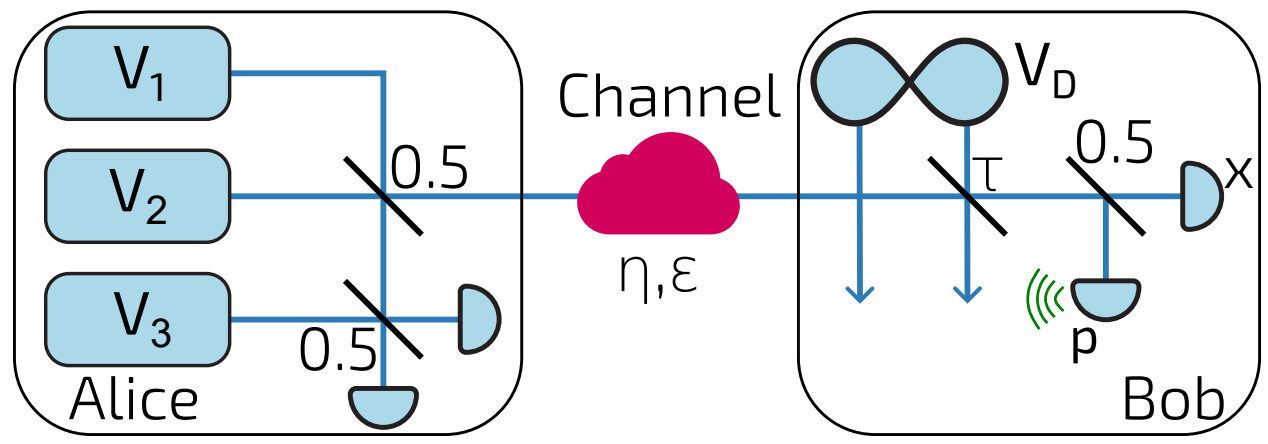

Figure S1: **Equivalent entanglement based protocol**

The equivalent entanglement-based (EB) model is illustrated in Fig. S1, where Alice holds the purification consisting of three single-mode squeezers, each with distinct squeezing parameters (*27, 28*). If Alice generates a pure single-mode squeezed states with the squeezing variance V_S and displaces them with Gaussian statistics with a modulation variance V_M in both quadratures, then the ensemble sent to Bob will have variances of $V_M + V_S$ in the x-quadrature and $V_M + 1/V_S$ in the p-quadrature. The variances of the three single-mode squeezers in Fig. S1 are sets as (*27*):

$$\begin{aligned} V_{1,2} &= V_S + V_M \pm \sqrt{\frac{(V_S + V_M)\,(V_M + V_S V_M (V_S + V_M))}{1 + V_S V_M}} \\ V_3 &= \frac{V_S^2 V_M (V_S + V_M)}{V_M (1 + V_S V_M)} \end{aligned} \tag{S1}$$

If we can consider a noisy squeezed state with the variance in the squeezed quadrature V_S, the variance in the anti-squeezed will be $V_S + \Delta V_{AN}$. Here, ΔV_{AN} represents the anti-squeezed noise, which is assumed to be trusted and under Alice's control. For a detailed analysis of scenarios involving untrusted anti-squeezed noise, readers can refer to Ref. (*28*). When modulating these noisy squeezed states, the variance of the states coming out of the channel will be $V_M + V_S$ and $V_M + 1/V_S + \Delta V_{AN}$. This added noise was assumed to be trusted and was modeled as extra modulation in the anti-squeezed quadrature (*28*).

The thermal channel, presumed to be under Eve's control, is characterized by its efficiency η and excess noise ϵ. Imperfect detection is modeled by overlapping the mode with another TMSV with a variance V_D on a beam splitter with the transmittance of τ after the channel. Bob then performs a heterodyne measurement on the received mode. Alice and Bob do not use the anti-squeezed quadrature for key generation but measurements in this quadrature are important for parameter estimation and system performance monitoring. In our protocol, Bob performs balanced

heterodyne detection on the received mode and publicly announces the results of the anti-squeezing measurements (see Section II.B in the main text or Ref. (*28*) for further details).

Parameter estimation

Parameter estimation is a crucial part of CV-QKD which allows the trusted parties to evaluate the information leakage from the untrusted channel. The channel is characterized by two parameters: the channel transmittance and the excess noise. After detection, the excess noise in conventional coherent states systems can be calculated with the conditional variance by

$$u = V_B - C_{AB}^2 - t - 1 \ , \tag{S2}$$

where V_B is the variance of the received signal from Bob normalized to shot noise, C_{AB}^2 is the normalized covariance between Alice and Bob at the receiver side, t is the trusted electronic noise of the detector and the shot noise, or vacuum state noise variance is normalized to 1. However, in the case of modulated squeezed state QKD the beautiful "1" in Eq. S2 from the vacuum states is replaced by the respective variances of the squeezed and anti-squeezed noise of the squeezed state scaled by the transmittance of the channel. Since the channel is fully under Eve's control, this noise floor cannot be directly measured as this would lead to a security loophole where the eavesdropper can change it, leading to a misestimation of the excess noise. To circumvent this, our protocol employs a procedure to estimate the noise floor based on the squeezing and anti-squeezing levels at the beginning of the channel established through B2B measurement along with the estimated channel efficiency.

In the B2B measurement, we measure a noisy squeezed state with an imperfect detector. Without loss of generality, we assume in the following that the generated states are squeezed in the X and anti-squeezed in the P quadrature. The variances measured in both quadratures read:

$$\begin{aligned} V_X^{\text{b2b}} &= V_{\text{sqz}}^{\text{b2b}} + t \\ V_P^{\text{b2b}} &= V_{\text{anti-sqz}}^{\text{b2b}} + \frac{\tau}{2}\Delta V_{AN} + t \end{aligned} \tag{S3}$$

where V_{sqz}^{b2b} is the measured squeezing variance, t is the electronic noise, and τ is the detection efficiency. The factor of $1/2$ comes from the heterodyne detection. Here, the noisy squeezed states were modeled by the pure squeezed states ($V_{anti-sqz}^{pure} = 1/V_{sqz}$) with added the anti-squeezed noise

ΔV_{AN}. The pure squeezing can be calculated by:

$$V_{sqz}^{\text{pure}} = 1 - \left[1 - (V_X^{\text{b2b}} - t)\right] / \left(\frac{\tau}{2}\right) \tag{S4}$$

In the anti-squeezed quadrature, we separate the anti-squeeze variance into the contribution from the pure anti-squeezed and the anti-squeezed noise ΔV_{AN}:

$$\begin{aligned} V_{anti-sqz}^{\text{b2b}} &= 1 + \frac{\tau}{2}\left(V_{\text{sqz}}^{-1} - 1\right) \\ \Delta V_{AN} &= (V_P^{\text{b2b}} - V_{\text{anti-sqz}}^{\text{b2b}} - t)/\left(\frac{\tau}{2}\right) \end{aligned} \tag{S5}$$

The PM covariance matrix after detection reads:

$$\gamma_{PM}^{RX} = \begin{bmatrix} V_M & 0 & \sqrt{\frac{\tau\eta}{2}}V_M & 0 \\ 0 & V_M & 0 & \sqrt{\frac{\tau\eta}{2}}V_M \\ \sqrt{\frac{\tau\eta}{2}}V_M & 0 & V_{\text{Rx}}^X & 0 \\ 0 & \sqrt{\frac{\tau\eta}{2}}V_M & 0 & V_{\text{Rx}}^P \end{bmatrix} \tag{S6}$$

where:

$$\begin{aligned} V_{\text{Rx}}^X &= \frac{\tau\eta}{2}V_M + u_x + t + V_{\text{sqz}}^{\text{Rx}} \\ V_{\text{Rx}}^P &= \frac{\tau\eta}{2}V_M + u_p + t + V_{\text{anti-sqz}}^{\text{Rx}} + \frac{\tau\eta}{2}\Delta V_{AN} \end{aligned} \tag{S7}$$

with η being the channel transmission efficiency, $V_{\text{sqz}}^{\text{Rx}}$ and $V_{\text{anti-sqz}}^{\text{Rx}}$ the squeezing and anti-squeezing variance after detection which can be calculated via:

$$\begin{aligned} V_{\text{sqz}}^{\text{Rx}} &= 1 - \frac{\tau\eta}{2}\left(1 - V_{\text{sqz}}^{\text{pure}}\right) \\ V_{\text{anti-sqz}}^{\text{Rx}} &= 1 + \frac{\tau\eta}{2}\left(V_{\text{anti-sqz}}^{\text{pure}} - 1\right) \end{aligned} \tag{S8}$$

Note that instead of the noise floor of 1, right now the noise floor in the X and P quadrature are $V_{\text{sqz}}^{\text{Rx}}$ and $V_{\text{anti-sqz}}^{\text{Rx}} + \frac{\tau\eta}{2}\Delta V_{AN}$, respectively. Thus, the excess noise after detection can be estimated as:

$$\begin{aligned} u_X &= V_X^{\text{det}} - \frac{\tau\eta}{2}V_M - V_{\text{sqz}}^{\text{Rx}} - t \\ &= V_X^{\text{det}} - C_{AB}^2 - V_{\text{sqz}}^{\text{Rx}} - t \\ u_P &= V_P^{\text{det}} - \frac{\tau\eta}{2}V_M - V_{\text{anti-sqz}}^{\text{Rx}} - \frac{\tau\eta}{2}\Delta V_{AN} - t \\ &= V_P^{\text{det}} - C_{AB}^2 - V_{\text{anti-sqz}}^{\text{Rx}} - \frac{\tau\eta}{2}\Delta V_{AN} - t \end{aligned} \tag{S9}$$

Parameters for information reconciliation

For the information reconciliation, we employ a MET-LDPC code with a code rate of 0.02 and the codeword length $n = 819200$ (*46*). To investigate the performance of the CV-QKD protocols across different β, we adopted a rate-adaptive reconciliation scheme. We used the puncturing technique to increase the code rate (*68*). The puncturing length p and the reconciliation efficiency β for the coherent states protocol and the squeezed-state protocol are detailed in Table S1 and Table S2 respectively.

MI (bits/quadrature)	R	Code length	p	β
0.0502	0.02	819200	463483	0.918

Table S1: **Parameters used for error correction in coherent-state protocol.**

MI (bits/quadrature)	R	Code length	p	β
0.0516	0.02	819200	472700	0.917
0.0516	0.02	819200	462162	0.890
0.0516	0.02	819200	425162	0.806

Table S2: **Parameters used for error correction in squeeze-state protocol.**

Parameters for excess noise tolerance investigation

Parameter estimation and experimental results for the investigation of excess noise tolerance for coherent states and squeezed-state protocol. V_{sqz} is the estimated squeezing variance before detection, ΔV_{AN} is the anti-squeezed noise variance, V_{mod} is the modulation variance, ξ_x and ξ_p are the excess noise in the two quadrature referred to channel input, η is the channel efficiency, t is the trusted detection noise referred to channel output. All the variances are calculated in SNU. The figure-of-merit: the SKF is calculated in bits/symbol. Table S3 and Table S4 showed the estimated parameters and the calculated SKF after 20 km and 30 km respectively.

	V_{sqz} (SNU)	ΔV_{AN} (SNU)	V_M (SNU)	ϵ_x (SNU)	ϵ_p (SNU)	η	V_D (SNU)	SKF
Coh.			1.067	0.0195	0.0195	0.407	1.0731	0.0531
				0.062	0.059	0.408	1.07	0.0132
				0.084	0.083	0.416	1.07	0
Sqz.	0.427	3.119		0.072	0.107	0.413	1.07	0.0208
				0.117	0.092	0.410	1.07	0.0167
				0.115	0.106	0.414	1.07	0.0178

Table S3: **Parameter estimation and experimental results for the 20 km channel.** The parameters were estimated from 10^8 quantum symbols.

Information-theoretic origin of squeezed states protocol advantage

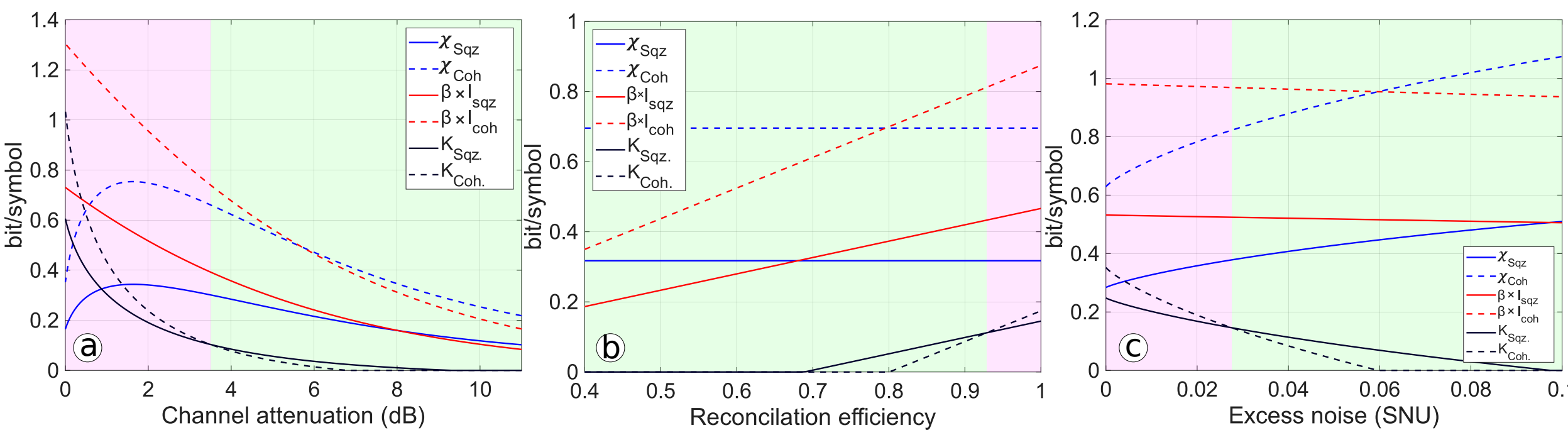

Figure S2: **Theoretical calculation of information per symbol in asymptotic regime**: Mutual information (I_{sqz} and I_{coh}), Holevo information (χ_{sqz} and χ_{coh}) and secret key rate (K_{sqz} and K_{coh}), when varying (a) channel attenuation, (b) reconciliation efficiency and (c) excess noise. The green regions show where squeezed states outperform coherent states protocol.

When considering cases where *both* coherent states and squeezed states protocols utilize a *single quadrature* for key generation, the advantage of squeezed states can be readily explained by the increase in mutual information and the reduction of Holevo information. Previous studies have thoroughly investigated scenarios involving homodyne detection and uni-dimensional protocols (*15, 25, 27*). In particular, Ref. (*15*) demonstrated that the intuitive advantage of squeezed states lies in the reduction of Holevo information, which can even be completely eliminated in the case

	V_{sqz} (SNU)	ΔV_{AN} (SNU)	V_M (SNU)	ϵ_x (SNU)	ϵ_p (SNU)	η	V_D (SNU)	SKF
Coh.			1.461	0.057	0.061	0.276	1.07	0.0074
				0.080	0.081	0.283	1.07	0
				0.104	0.103	0.274	1.07	0
Sqz.	0.416	2.714		0.017	0.063	0.291	1.07	0.0315
				0.081	0.112	0.292	1.07	0.0162
				0.103	0.155	0.291	1.07	0.0039

Table S4: **Parameter estimation and experimental results for the 30 km channel.** The parameters were estimated from 10^8 quantum symbols.

of a purely lossy channel. However, the requirement for homodyne detection at the channel output increases implementation complexity and limits the protocol's practicality. However, in this study, we compare two protocols: a conventional *coherent states* protocol where secret keys are generated using *both quadratures*, and a *squeezed-state protocol* with heterodyne detection where secret keys are generated using *only the squeezed quadrature*. Due to these differing configurations, explaining the inherent advantages of squeezed states becomes more nuanced. The secret key rate is determined by two nonlinear quantities: mutual information and Holevo information (Figure S2). It is the interplay between these two functions and the reconciliation efficiency that provides the advantage of squeezed states.

While the coherent states protocol yields higher mutual information by generating keys in both quadratures (providing almost twice the information), the Holevo information scales highly nonlinearly with variations in channel efficiency and excess noise (Figures S2a and S2c). When the reconciliation efficiency decreases, the Holevo information remains unaffected, but the effective mutual information between Alice and Bob decreases (Figure S2b). Here, due to the better signal-to-noise ratio provided by the squeezed states, the mutual information decreases more slowly than in the coherent states case. Conversely, an increase in excess noise reduces the mutual information similarly in both protocols, but the Holevo information increases much more drastically in the coherent states case. Ultimately, these factors lead to a cut-off point where squeezed states outperform coherent states (Figures S2b and S2c).

In fact, early developments in CV-QKD has considered measuring both quadratures of the squeezed signal state (*45*), and have noted increased key rate in comparison with the coherent-state protocol. Although, the protocol used in our work differs (since we perform announcement of the non-signal quadrature) the rationale remains the same - heterodyne measurements introduce additional (trusted vacuum) noise that effectively counteracts the excess noise added in the channel. Latter is also known as ”fighting noise with noise” (*69*). Interestingly, the volume of such noise can be optimized, which is considered in previous theoretical works (*28, 45*).

Squeezed states versus coherent states CV-QKD over long distance channel

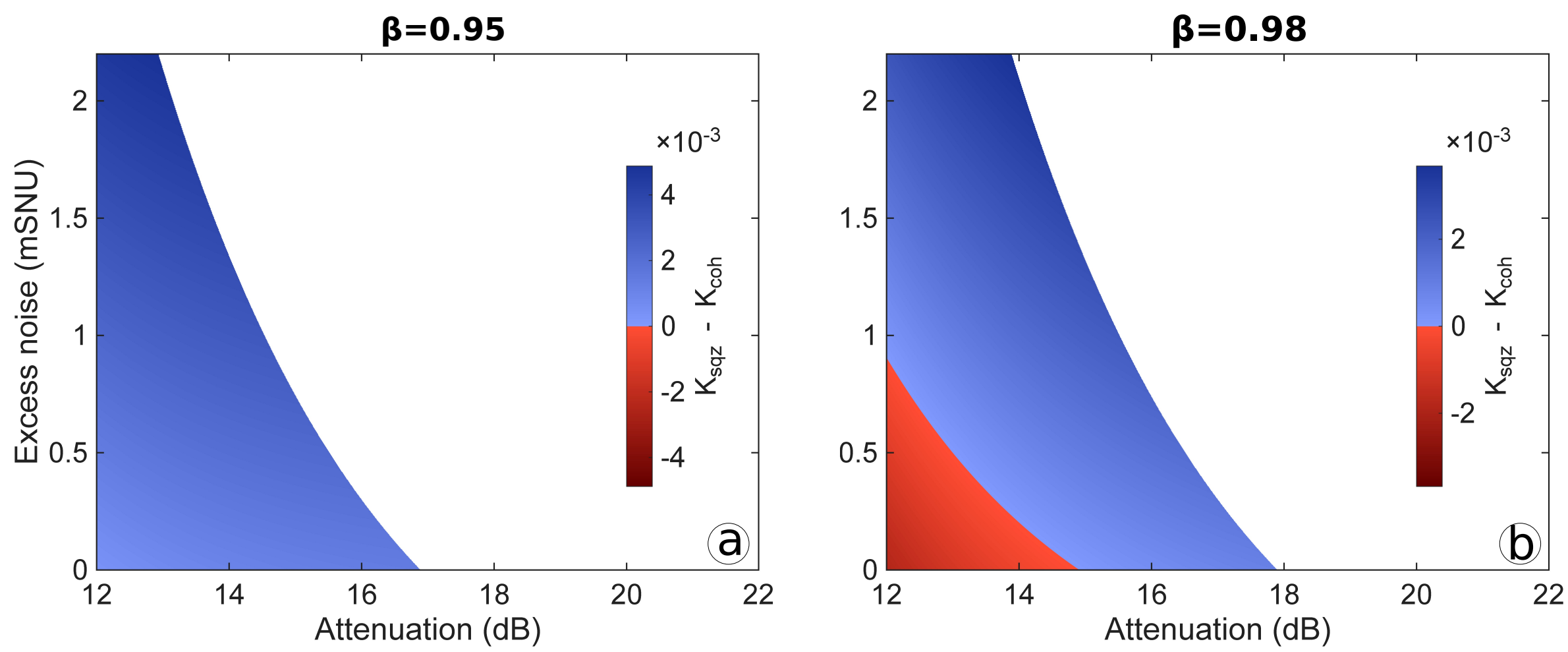

Figure S3: **Difference in the secure key rate between squeezed-state and coherent-state protocols** ($K_{qz} - K_{coh}$, bit/symbol). The blue region indicates regimes where squeezed states outperform coherent states, the red region denotes where coherent states are superior, and the white region corresponds to parameters where neither protocol can generate a secure key. Excess noise is considered at the *end of the channel*.

Figure S3 shows the finite-size key rate difference between squeezed states and coherent states ($K_{qz} - K_{coh}$). The x-axis shows the channel attenuation, corresponding to approximately 80 km to 150 km of ultra-low-loss fiber, while the y-axis shows the excess noise at the channel output. In both protocols, the modulation variance was set to $V_{mod} = 5$ SNU, the trusted detection efficiency was $\tau = 0.68$, the trusted noise was $V_D = 0.07$ and the block size is 10^9. The squeezing level was set to a realistic 3 dB with 3 SNU of anti-squeezed noise. Squeezed states continue to show

superior performance even at longer distances. With a reconciliation efficiency of $\beta = 0.95$, the squeezed-state protocol always outperforms coherent states (Figure S3a. Assuming a highly efficient reconciliation process ($\beta = 0.98$), the coherent-state protocol yields a higher finite-size key rate only under conditions of low excess noise (< 1 mSNU at the channel output) and channel attenuations below 15 dB (Bottom left part of Figure S3b. Thus, this analysis demonstrates that squeezed states not only maintain their performance advantage over long distance channels but also serve as a vital resource for extending the maximum transmission reach of CV-QKD systems.

REFERENCES

1. S. Pirandola, U. L. Andersen, L. Banchi, M. Berta, D. Bunandar, R. Colbeck, D. Englund, T. Gehring, C. Lupo, C. Ottaviani, J. L. Pereira, M. Razavi, J. Shamsul Shaari, M. Tomamichel, V. C. Usenko, G. Vallone, P. Villoresi, P. Wallden, Advances in quantum cryptography. *Adv. Opt. Photonics* **12**, 1012–1236 (2020).

2. V. C. Usenko, A. Acín, R. Alléaume, U. L. Andersen, E. Diamanti, T. Gehring, A. A. E. Hajomer, F. Kanitschar, C. Pacher, S. Pirandola, V. Pruneri, Continuous-variable quantum communication. *Rev. Mod. Phys.* **98**, 015003 (2026).

3. N. Jain, H.-M. Chin, H. Mani, C. Lupo, D. S. Nikolic, A. Kordts, S. Pirandola, T. B. Pedersen, M. Kolb, B. Ömer, C. Pacher, T. Gehring, U. L. Andersen, Practical continuous-variable quantum key distribution with composable security. *Nat. Commun.* **13**, 4740 (2022).

4. A. A. E. Hajomer, I. Derkach, N. Jain, H.-M. Chin, U. L. Andersen, T. Gehring, Long-distance continuous-variable quantum key distribution over 100-Km fiber with local local oscillator. *Sci. Adv.* **10**, eadi9474 (2024).

5. Y. Zhang, Z. Chen, S. Pirandola, X. Wang, C. Zhou, B. Chu, Y. Zhao, B. Xu, S. Yu, H. Guo, Long-distance continuous-variable quantum key distribution over 202.81 Km of fiber. *Phys. Rev. Lett.* **125**, 010502 (2020).

6. A. A. E. Hajomer, C. Bruynsteen, I. Derkach, N. Jain, A. Bomhals, S. Bastiaens, U. L. Andersen, X. Yin, T. Gehring, Continuous-variable quantum key distribution at 10 GBaud using an integrated photonic-electronic receiver. *Optica* **11**, 1197–1204 (2024).

7. T. Morimae, Continuous-variable blind quantum computation. *Phys. Rev. Lett.* **109**, 230502 (2012).

8. N. C. Menicucci, P. van Loock, M. Gu, C. Weedbrook, T. C. Ralph, M. A. Nielsen, Universal quantum computation with continuous-variable cluster states. *Phys. Rev. Lett.* **97**, 110501 (2006).

9. Z.-H. Liu, R. Brunel, E. E. B. Østergaard, O. Cordero, S. Chen, Y. Wong, J. A. H. Nielsen, A. B. Bregnsbo, S. Zhou, H.-Y. Huang, C. Oh, L. Jiang, J. Preskill, J. S. Neergaard-Nielsen, U. L. Andersen, Quantum learning advantage on a scalable photonic platform. *Science* **389**, 1332–1335 (2025).

10. J. A. H. Nielsen, J. S. Neergaard-Nielsen, T. Gehring, U. L. Andersen, Deterministic quantum phase estimation beyond N00N states. *Phys. Rev. Lett.* **130**, 123603 (2023).

11. X. Guo, C. R. Breum, J. Borregaard, S. Izumi, M. V. Larsen, T. Gehring, M. Christandl, J. S. Neergaard-Nielsen, U. L. Andersen, Distributed quantum sensing in a continuous-variable entangled network. *Nat. Phys.* **16**, 281–284 (2020).

12. F. Furrer, T. Gehring, C. Schaffner, C. Pacher, R. Schnabel, S. Wehner, Continuous-variable protocol for oblivious transfer in the noisy-storage model. *Nat. Commun.* **9**, 1450 (2018).

13. L. S. Madsen, V. C. Usenko, M. Lassen, R. Filip, U. L. Andersen, Continuous variable quantum key distribution with modulated entangled states. *Nat. Commun.* **3**, 1083 (2012).

14. T. Gehring, V. Händchen, J. Duhme, F. Furrer, T. Franz, C. Pacher, R. F. Werner, R. Schnabel, Implementation of continuous-variable quantum key distribution with composable and one-sided-device-independent security against coherent attacks. *Nat. Commun.* **6**, 8795 (2015).

15. C. S. Jacobsen, L. S. Madsen, V. C. Usenko, R. Filip, U. L. Andersen, Complete elimination of information leakage in continuous-variable quantum communication channels. *npj Quantum Inf.* **4**, 32 (2018).

16. G. Chesi, S. Olivares, M. G. A. Paris, Squeezing-enhanced phase-shift-keyed binary communication in noisy channels. *Phys. Rev. A* **97**, 032315 (2018).

17. A. Walsh, L. Conlon, B. Shajilal, O. Erkilic, J. Janousek, S. Assad, J. Zhao, P. K. Lam, All-Gaussian state discrimination beyond the coherent helstrom bound. arXiv:2510.20096 [quant-ph] (2025).

18. R. E. S. Polkinghorne, T. C. Ralph, Continuous variable entanglement swapping. *Phys. Rev. Lett.* **83**, 2095–2099 (1999).

19. A. Furusawa, J. L. Sørensen, S. L. Braunstein, C. A. Fuchs, H. J. Kimble, E. S. Polzik, Unconditional quantum teleportation. *Science* **282**, 706–709 (1998).

20. J. Zhao, H. Jeng, L. O. Conlon, S. Tserkis, B. Shajilal, K. Liu, T. C. Ralph, S. M. Assad, P. K. Lam, Enhancing quantum teleportation efficacy with noiseless linear amplification. *Nat. Commun.* **14**, 4745 (2023).

21. F. Grosshans, P. Grangier, Continuous variable quantum cryptography using coherent states. *Phys. Rev. Lett.* **88**, 057902 (2002).

22. C. Weedbrook, A. M. Lance, W. P. Bowen, T. Symul, T. C. Ralph, P. K. Lam, Quantum cryptography without switching. *Phys. Rev. Lett.* **93**, 170504 (2004).

23. N. J. Cerf, M. Lévy, G. V. Assche, Quantum distribution of Gaussian keys using squeezed states. *Phys. Rev. A* **63**, 052311 (2001).

24. M. S. Winnel, N. Hosseinidehaj, T. C. Ralph, Minimization of information leakage in continuous-variable quantum key distribution. *Phys. Rev. A* **104**, 012411 (2021).

25. I. Derkach, V. C. Usenko, R. Filip, Squeezing-enhanced quantum key distribution over atmospheric channels. *New J. Phys.* **22**, 053006 (2020).

26. N. Hosseinidehaj, M. S. Winnel, T. C. Ralph, Simple and loss-tolerant free-space quantum key distribution using a squeezed laser. *Phys. Rev. A* **105**, 032602 (2022).

27. V. C. Usenko, R. Filip, Squeezed-state quantum key distribution upon imperfect reconciliation. *New J. Phys.* **13**, 113007 (2011).

28. A. n. Oruganti, I. Derkach, R. Filip, V. C. Usenko, Continuous-variable quantum key distribution with noisy squeezed *states Sci. Technol.* **10**, 025023 (2025).

29. J. Arnbak, C. S. Jacobsen, R. B. Andrade, X. Guo, J. S. Neergaard-Nielsen, U. L. Andersen, T. Gehring, Compact, low-threshold squeezed light source. *Opt. Express* **27**, 37877–37885 (2019).

30. Y. Zhang, M. Menotti, K. Tan, V. D. Vaidya, D. H. Mahler, L. G. Helt, L. Zatti, M. Liscidini, B. Morrison, Z. Vernon, Squeezed light from a nanophotonic molecule. *Nat. Commun.* **12**, 2233 (2021).

31. R. Nehra, R. Sekine, L. Ledezma, Q. Guo, R. M. Gray, A. Roy, A. Marandi, Few-cycle vacuum squeezing in nanophotonics. *Science* **377**, 1333–1337 (2022).

32. T. Park, H. Stokowski, V. Ansari, S. Gyger, K. K. S. Multani, O. T. Celik, A. Y. Hwang, D. J. Dean, F. Mayor, T. P. McKenna, M. M. Fejer, A. Safavi-Naeini, Single-mode squeezed-light generation and tomography with an integrated optical parametric oscillator. *Sci. Adv.* **10**, eadl1814 (2024).

33. T. N. Arge, S. Jo, H. Q. Nguyen, F. Lenzini, E. Lomonte, J. A. H. Nielsen, R. R. Domeneguetti, J. S. Neergaard-Nielsen, W. Pernice, T. Gehring, U. L. Andersen, Demonstration of a squeezed light source on thin-film lithium niobate with modal phase matching. *Opt. Quantum* **3**, 467–473 (2025).

34. J.-Z. Huang, C. Weedbrook, Z.-Q. Yin, S. Wang, H.-W. Li, W. Chen, G.-C. Guo, Z.-F. Han, Quantum hacking of a continuous-variable quantum-key-distribution system using a wavelength attack. *Phys. Rev. A* **87**, 062329 (2013).

35. P. Jouguet, S. Kunz-Jacques, E. Diamanti, Preventing calibration attacks on the local oscillator in continuous-variable quantum key distribution. *Phys. Rev. A* **87**, 062313 (2013).

36. I. Suleiman, J. A. H. Nielsen, X. Guo, N. Jain, J. Neergaard-Nielsen, T. Gehring, U. L. Andersen, 40 Km fiber transmission of squeezed light measured with a real local oscillator. *Quantum Sci. Technol.* **7**, 045003 (2022).

37. I. Suleiman, “Long-Distance Continuous Variable Quantum Communication,” thesis, Technical University of Denmark (2022).

38. H.-M. Chin, N. Jain, U. L. Andersen, D. Zibar, T. Gehring, Digital synchronization for continuous-variable quantum key distribution. *Quantum Sci. Technol.* **7**, 045006 (2022).

39. H.-M. Chin, N. Jain, D. Zibar, U. L. Andersen, T. Gehring, Machine learning aided carrier recovery in continuous-variable quantum key distribution. *npj Quantum Inf.* **7**, 20 (2021).

40. Z.-K. Zhang, W.-Q. Liu, J. Qi, C. He, P. Huang, Automatic phase compensation of a continuous-variable quantum-key-distribution system via deep learning. *Phys. Rev. A* **107**, 062614 (2023).

41. Ö. Erkılıç, B. Shajilal, L. O. Conlon, A. Walsh, A. Das, S. Kish, T. Symul, P. K. Lam, S. M. Assad, J. Zhao, Enhanced continuous-variable quantum key distribution protocol via adaptive signal processing. *Commun. Phys.* **8**, 406 (2025).

42. H. Q. Nguyen, I. Derkach, A. A. E. Hajomer, H.-M. Chin, A. n. Oruganti, U. L. Andersen, V. Usenko, T. Gehring, Digital reconstruction of squeezed light for quantum information processing. *npj Quantum Information* **11**, 71 (2025).

43. T. C. Ralph, Continuous variable quantum cryptography. *Phys. Rev. A* **61**, 010303 (1999).

44. R. García-Patrón, N. J. Cerf, Continuous-variable quantum key distribution protocols over noisy channels. *Phys. Rev. Lett.* **102**, 130501 (2009).

45. H. Mani, T. Gehring, P. Grabenweger, B. Ömer, C. Pacher, U. L. Andersen, Multiedge-type low-density parity-check codes for continuous-variable quantum key distribution. *Phys. Rev. A* **103**, 062419 (2021).

46. W.-B. Liu, C.-L. Li, Y.-M. Xie, C.-X. Weng, J. Gu, X.-Y. Cao, Y.-S. Lu, B.-H. Li, H.-L. Yin, Z.-B. Chen, Homodyne detection quadrature phase shift keying continuous-variable quantum key distribution with high excess noise tolerance. *PRX Quantum* **2**, 040334 (2021).

47. S. Q. Ng, F. Kanitschar, G. Zhang, C. Wang, Gigabit-rate quantum key distribution on integrated photonic chips. arXiv:2504.08298 [quant-ph] (2025).

48. H. Yin, P. Huang, Z. Zhou, T. Wang, X. Jiang, G. Zeng, All-day free-space quantum key distribution with thermal source towards quantum secure communications for unmanned vehicles. *npj Quantum Inf.* **11**, 134 (2025).

49. X.-T. Zheng, Q.-F. Zhang, J. Ling, G.-C. Guo, Z.-F. Han, Free-space continuous-variable quantum key distribution under high background noise. *npj Quantum Inf.* **11**, 52 (2025).

50. S.-K. Liao, W.-Q. Cai, W.-Y. Liu, L. Zhang, Y. Li, J.-G. Ren, J. Yin, Q. Shen, Y. Cao, Z.-P. Li, F.-Z. Li, X.-W. Chen, L.-H. Sun, J.-J. Jia, J.-C. Wu, X.-J. Jiang, J.-F. Wang, Y.-M. Huang, Q. Wang, Y.-L. Zhou, L. Deng, T. Xi, L. Ma, T. Hu, Q. Zhang, Y.-A. Chen, N.-L. Liu, X.-B. Wang, Z.-C. Zhu, C.-Y. Lu, R. Shu, C.-Z. Peng, J.-Y. Wang, J.-W. Pan, Satellite-to-ground quantum key distribution. *Nature* **549**, 43–47 (2017).

51. Y. Li, W.-Q. Cai, J.-G. Ren, C.-Z. Wang, M. Yang, L. Zhang, H.-Y. Wu, L. Chang, J.-C. Wu, B. Jin, H.-J. Xue, X.-J. Li, H. Liu, G.-W. Yu, X.-Y. Tao, T. Chen, C.-F. Liu, W.-B. Luo, J. Zhou, H.-L. Yong, Y.-H. Li, F.-Z. Li, C. Jiang, H.-Z. Chen, C. Wu, X.-H. Tong, S.-J. Xie, F. Zhou, W.-Y. Liu, Y. Ismail, F. Petruccione, N.-L. Liu, L. Li, F. Xu, Y. Cao, J. Yin, R. Shu, X.-B. Wang, Q. Zhang, J.-Y. Wang, S.-K. Liao, C.-Z. Peng, J.-W. Pan, Microsatellite-based real-time quantum key distribution. *Nature* **640**, 47–54 (2025).

52. C. Peuntinger, B. Heim, C. R. Müller, C. Gabriel, C. Marquardt, G. Leuchs, Distribution of squeezed states through an atmospheric channel. *Phys. Rev. Lett.* **113**, 060502 (2014).

53. M. Hillery, V. Bužek, A. Berthiaume, Quantum secret sharing. *Phys. Rev. A* **59**, 1829–1834 (1999).

54. R. Cleve, D. Gottesman, H.-K. Lo, How to share a quantum secret. *Phys. Rev. Lett.* **83**, 648–651 (1999).

55. C. Schmid, P. Trojek, M. Bourennane, C. Kurtsiefer, M. Z. Żukowski, H. Weinfurter, Experimental single qubit quantum secret sharing. *Phys. Rev. Lett.* **95**, 230505 (2005).

56. Y.-A. Chen, A.-N. Zhang, Z. Zhao, X.-Q. Zhou, C.-Y. Lu, C.-Z. Peng, T. Yang, J.-W. Pan, Experimental quantum secret sharing and third-man quantum cryptography. *Phys. Rev. Lett.* **95**, 200502 (2005).

57. Y.-R. Xiao, H.-L. Yin, W.-J. Hua, X.-Y. Cao, Z.-B. Chen, Experimental efficient source-independent quantum secret sharing against coherent attacks. *Phys. Rev. Lett.* **135**, 150801 (2025).

58. A. M. Lance, T. Symul, W. P. Bowen, B. C. Sanders, P. K. Lam, Tripartite quantum state sharing. *Phys. Rev. Lett.* **92**, 177903 (2004).

59. S. Liu, Z. Lu, P. Wang, Y. Tian, X. Wang, Y. Li, Experimental demonstration of multiparty quantum secret sharing and conference key agreement. *npj Quantum Inf.* **9**, 92 (2023).

60. Q. Liao, Z. Fei, L. Huang, X. Fu, Practical continuous-variable quantum secret sharing using local local oscillator. *Commun. Phys.* **8**, 138 (2025).

61. L. O. Conlon, B. Shajilal, A. Walsh, J. Zhao, J. Janousek, P. K. Lam, S. M. Assad, Verifying the security of a continuous variable quantum communication protocol via quantum metrology. *npj Quantum Inf.* **10**, 35 (2024).

62. A. A. E. Hajomer, N. Jain, H. Mani, H.-M. Chin, U. L. Andersen, T. Gehring, Modulation leakage-free continuous-variable quantum key distribution. *npj Quantum Inf.* **8**, 136 (2022).

63. Z. Chen, X. Wang, S. Yu, Z. Li, H. Guo, Continuous-mode quantum key distribution with digital signal processing. *npj Quantum Inf.* **9**, 28 (2023).

64. B. Qi, P. Lougovski, R. Pooser, W. Grice, M. Bobrek, Generating the local oscillator "Locally" in continuous-variable quantum key distribution based on coherent detection. *Phys. Rev. X* **5**, 041009 (2015).

65. DTU Computing Center Resources, DTU Computing Center (2024).

66. A. Leverrier, Composable security proof for continuous-variable quantum key distribution with coherent states. *Phys. Rev. Lett.* **114**, 070501 (2015).

67. X. Wang, Y. Zhang, S. Yu, B. Xu, Z. Li, H. Guo, Efficient rate-adaptive reconciliation for continuous-variable quantum key distribution. *Quant. Inf. Comput.* **17**, 1123–1134 (2017).

68. V. C. Usenko, R. Filip, Trusted noise in continuous-variable quantum key distribution: A threat and a defense. *Entropy* **18**, 20 (2016).
